# Geographical Pathology

**Published:** 1893-02

**Authors:** 


GEOGRAPHICAL PATHOLOGY: An Inquiry into the Geographical Distribution of the Infective and Climatic Diseases. In two volumes, by ANDREW DAVIDSON, M. D., F. R. C. P., Ed., Late Visiting and Superintending Surgeon, Civil Hospital, and Professor of Chemistry, Royal College, Mauritius. Europe, Northern and Western Asia, India, Ceylon, and Burmah. Vols. I. and II. New York: D. Appleton & Co. 1892.

This work occupies a somewhat novel field in the literature of medicine. Its object, as stated by the author, is to sketch the geographical distribution of infective and climatic diseases, and to trace the influence of temperature, rainfall, altitude, and soil-conditions on their prevalence, character, and epidemic spread.

In the pursuit of this object, Davidson presents a study of these diseases in the various subdivisions of Europe, Asia, Africa, and the Western Hemisphere. He has thus, in his observations, given to the profession a reference book which will tell, with a reasonable degree of accuracy, the prevailing diseases of the various countries of the world at their probable season, as well as information on the subject of climate, moisture, dryness, and temperature. The work is rather one for reference than for consecutive reading, but it is also one that must be examined with some detail in order to understand the valuable relations it has to the study of geographical disease prevalence.

The wide extent of territory covered by the United States makes it a favorable field for the study on a large scale of the effects of temperature, rainfall, altitude, soil, and social conditions on the health of the community ; and the peculiarity in this country is that one may include in such a study, side by side, the Caucasian, Indian, and Negro races. Davidson has availed himself of this opportunity, and gives a faithful setting forth of the bearings these several conditions have upon disease. Most of the observations contained in this volume are based on the tenth census, 1880, and so would appear to be about twelve years behind the conditions of the present. But, it must be remembered that for the purposes intended in a research of this sort, a decade can make very little difference in the ratios or in the climatological influences upon diseases. It is not probable, however, that a new edition will be demanded or even needful until the census of 1900 ; it then may be made to include such changes as increase of population and cultivation of soil in the West may entail.

